# Effects of secretin gene knockout on the diversity, composition, and function of gut microbiota in adult male mice

**DOI:** 10.3389/fcimb.2023.1257857

**Published:** 2023-12-13

**Authors:** Fengwei Zhang, Zhengyi Tao, Congjia Chen, Billy Kwok Chong Chow

**Affiliations:** School of Biological Sciences, The University of Hong Kong, Hong Kong, Hong Kong SAR, China

**Keywords:** secretin, gut microbiota, metabolism, gut hormone, 16S rRNA amplicon sequencing

## Abstract

The gut microbiota plays a vital role in maintaining gastrointestinal homeostasis, however, whether it is influenced by gut hormones remains unknown. Secretin is a well-known gastrointestinal hormone produced by enteroendocrine S cells. This study utilized 16S rRNA amplicon sequencing to characterize the effect of SCT deficiency on the gut microbiota. Our results show that systemic SCT knockout alters the composition and abundance of the mouse gut microbiota but does not affect fecal short-chain fatty acids and lipids concentrations. At the genus level, the abundance of *Turicibacter*, *Bacteroides*, *Ruminococcu*, *Romboutsia*, *Asaccharobacter*, and *Parasutterella* increased in SCT^-/-^ mice, whereas the abundance of *Akkermansia* and *Escherichia* decreased. Functional prediction results showed that lack of SCT reduced the abundance of carbohydrate metabolism-related pathways but increased the abundance of linoleic acid metabolism and branched-chain amino acid degradation. Overall, systemic SCT knockout had only minor effects on gut microbiota composition and function in adult male mice fed a standard chow diet.

## Introduction

The gut microbiota is a complex microbial community located in the gastrointestinal (GI) tract that establishes a close symbiotic relationship with the host. Gut microbiota and GI hormones are two key components of the GI system that play important roles in regulating various physiological processes such as digestion, metabolism, and immune response (Clarke et al., 2014; Fukui et al., 2018; Sun et al., 2020; Woźniak et al., 2021). Previous studies show that the gut microbiota can regulate the production and secretion of GI hormones. For example, germ-free mice had decreased levels of serotonin (Wikoff et al., 2009) and increased levels of glucagon-like peptide-1 (GLP-1) (Wichmann et al., 2013) compared with conventional mice. Furthermore, the intake of prebiotics that promote the growth of *Lactobacillus* and *Bifidobacterium* reduces ghrelin secretion in obese humans (Parnell and Reimer, 2009). An evolution-oriented study hypothesizes that multiple enzymes related to GI hormone metabolism may have evolved from bacterial genes (Hume et al., 2017; Nicolucci et al., 2017). On the other hand, GI hormones also modulate the diversity and composition of the gut microbiota. For example, serotonin released by enterochromaffin cells is secreted not only into the intestinal submucosa but also into the intestinal lumen, which may lead to alterations in the gut microbiota (Patel, 2011). The influence of GI hormones on gut microbiota is critical for maintaining a healthy gut environment and normal physiological function. Understanding this role will benefit the development of therapeutics for various gut-related diseases.

Secretin (SCT) is a GI hormone produced by enteroendocrine S cells (Afroze et al., 2013). SCT regulates intestinal pH by inhibiting gastric acid secretion and stimulating bicarbonate production (Whitmore et al., 2000). Studies from different species have shown the presence of SCT-expressing cells and high-level SCT expression in the duodenum, small intestine, and colon (Polak et al., 1971; Van Ginneken and Weyns, 2004; Modvig et al., 2020; Liu et al., 2023). Early studies found that intravenous infusion of SCT exerts trophic effects on the gut of dogs and increased galactose uptake in the jejunum and ileum (Hughes et al., 1978). Our previous study showed that SCT receptor knockout led to intestinal lipid malabsorption (Sekar and Chow, 2014). These results suggest that SCT is essential for maintaining healthy gut function. However, the impact of SCT on gut microbiota composition and function remains unclear. In this study, 16S rRNA sequencing was used to compare the differences in gut microbiota between whole-body SCT knockout (SCT^-/-^) and wild-type (SCT^+/+^) littermates. By performing α and β diversity analysis, LEfSe analysis, and functional predictive analysis on the sequencing results, this study explored the effect of systemic SCT deficiency on the taxonomy and function of gut microbiota.

## Materials and methods

### Animals

All animal care and experimental procedures were carried out with the protocols approved by the Committee on the Use of Live Animals in Teaching and Research (CULATR) of the University of Hong Kong (protocol No. 5791-21). Animals were maintained in a facility accredited by the Association for the Assessment and Accreditation of Laboratory Animal Care International (AAALAC). SCT^-/-^ mice were previously generated (Lee et al., 2010) and have been used in recent studies (Zaw et al., 2019; Zhang et al., 2022; Liu et al., 2023). SCT^+/-^ male mice were backcrossed with female C57BL6/N (Charles River Laboratories, strain code: 027) mice to purify the mixed genetic background; each backcross was indicated by an increase in N number. All experiments were carried out using male mice. SCT^-/-^ and SCT^+/+^ littermates were obtained from heterozygous parents. All mice were housed in temperature-controlled and humidity-controlled rooms with a 12:12 h light: dark cycle with *ad libitum* access to standard rodent chow (0.3% Na; Test Diet, 5881) and water unless otherwise specified. Feces from 12-week-old SCT^+/+^ (n = 7) and SCT^-/-^ (n = 7) mice were collected on the same day at 10:00 am and then frozen at −80°C prior to DNA extraction.

### RNA isolation and real-time qPCR.

Total RNA was extracted from tissues using TRIzol™ Reagent (15596026, ThermoFisher) following the manufacturer’s instructions; 1 μg of total RNA was used to synthesize cDNA using the HiScript^®^III All-in-one RT SuperMix (R333-01, Vazyme biotech). An aliquot (1/5 vol) of the cDNA was then subjected to qPCR using the ChamQ SYBR qPCR Master Mix (Q411-02, Vazyme biotech) in a 96-well real-time PCR machine (7300 Real-Time PCR System, Applied Biosystems). Fold changes were calculated and determined using the 2^-ΔΔCt^ method and expression levels normalized to the average of the housekeeping genes 18S. The following primers were used: *18S* F: 5’-CTCTAGATAACCTCGGGCC-3′, R: 5′-GAACCCTGATTCCCCGTCA-3′; *Sct* F: 5’-GACCATGGAGCCTCCGCTG-3′, R: 5′-GGACAACCAATCCCTACTCC-3′.

### ELISA assays

Blood and tissue SCT were analyzed using an ELISA kit (EK-067-04, Phoenix Pharmaceuticals) following the manufacturer’s instructions. Total tissue protein was extracted using RIPA lysis buffer (89900, ThermoFisher), and protein concentrations were measured using a BCA Protein Assay Kit (P0011, Beyotime).

### Quantification of fecal lipids

Sufficient fecal pellets from 12-week-old SCT^+/+^/SCT^-/-^ mice (n = 10 for each group) were collected for analysis. Extraction of fecal lipids was performed as previously described (Hara and Radin, 1978). Lipids were extracted from feces with a 30-fold volume of chloroform/methanol (2:1, v/v), dried, and the dried residue dissolved in 5% Triton X-100 in isopropanol. Total cholesterol, triglyceride, and free fatty acid (FFA) concentrations were quantified using Biochemical Assay Kits (FUJIFILM Wako Chemicals) following the manufacturer’s instructions.

### Quantification of fecal short-chain fatty acids

Short-chain fatty acids (SCFAs) levels were determined in stool samples by gas chromatography-mass spectrometry (GC-MS) (7890A-5975C, Agilent Technologies) using a 30 m × 0.25 mm × 0.25 µm capillary column (HP1-MS, Agilent Technologies). Measurement of SCFAs was performed as previously described (Laurans et al., 2018) with slight modifications. Briefly, 30 mg of stool samples were suspended in 200 μL of 0.15 M NaOH solution. After the addition of the standards, each sample was acidified and then extracted with diethyl ether. Samples were stirred gently for 1 h and then centrifuged for 2 min (5000 rpm, 4°C). The organic layers were transferred into 1.5 mL glass vials and SCFAs were derivatized with 20 µL of tert-butyldimethylsilyl imidazole. Samples were incubated for 30 min at 60°C before analysis. The contents of SCFAs in the samples were calculated based on the standard curve.

### Gut microbiota DNA extraction and 16S rRNA amplicon sequencing

The total genomic DNA (gDNA) in each sample was extracted using a QIAamp PowerFecal Pro DNA Kit (51804, QIAGEN) according to the manufacturer’s instructions. The quality and mass of gDNA were determined by a Qubit fluorometer (ThermoFisher). 30ng of gDNA from each sample was used to amplify the V3–V4 region of 16S ribosomal RNA (rRNA). As primers of 16s V3-V4 region amplification, a set of 8-nucleotide barcodes was connected to the V3-V4 forward primer 338F (5’-ACTCCTACGGGAGGCAGCA-3’) and the reverse primer 806R (5’-GGACTACHVGGGTWTCTAAT-3’). PCR amplification was performed with 2 × Phanta Max Master Mix (P515-01, Vazyme) and S100 Thermal Cycler (Bio-Rad) under optimized conditions. The PCR product was subjected to the following circularization reaction: pre-denaturation (95°C, 3 min), followed by 25 cycles of denaturation (95°C), annealing (55°C), and extension (72°C) for 30 sec each, and finally extended to 72°C for 5 min. All PCR products were purified by Agencourt AMPure XP beads (A63880, Beckman Coulter), dissolved in elution buffer, and eventually labeled to finish DNA nanoball (DNB) library construction. Library size and concentration were detected by 2100 Bioanalyzer (Agilent). Qualified DNB libraries were sequenced on the DNBSEQ-G400 High-throughput Sequencing platform from BGI Group (Hong Kong, China).

### Bioinformatics analysis

The raw sequences were sorted into different samples according to the barcodes. Raw data were filtered by readfq (v1.0), and the primer and adapter contamination were removed by the cutadapt (v2.6), and low-quality (more than 20% base quality < Q20) reads were then removed by the quality control software iTools Fqtools fqcheck (v.0.25). The paired reads were assembled into a consensus sequence (i.e., the Tags) by the FLASH (v1.2.11) (Magoč and Salzberg, 2011) with parameters “–min-overlap 15 –max-mismatch-density 0.1”. The spliced Tags were then clustered into operational taxonomic units (OTUs) using the UPARSE in USEARCH (v7.0.1090) with a 97% similarity threshold. Chimeric sequences generated by PCR amplification were removed from OTU representative sequences using UCHIME (v4.2.40). OTUs were aligned against the database for taxonomic annotation by Ribosomal Database Project (RDP) classifier (v2.2) software with a confidence value of 0.6. OTUs with no annotation or non-bacteria annotation results were removed and the remaining OTUs were used for further analysis. The community richness index and community diversity index were calculated by the R “Vegan” package (Zhang et al., 2019a) to determine the alpha diversity. The beta diversity was calculated using the Bray-Curtis distance and visualized with principal coordinate analysis (PCoA) to find the differences in microbiota structure between groups. Taxonomies can annotate species information from phylum to species level. Linear Discriminant Analysis Effect Size (LEfSe) was applied to analyze the differences at each level (Segata et al., 2011). Based on the high-quality sequences, functional gene and Kyoto Encyclopedia of Genes and Genomes (KEGG) pathways were predicted by the Phylogenetic Investigation of Communities by Reconstruction of Unobserved States (PICRUSt2) (v2.3.0-b) (Langille et al., 2013).

### Statistical analysis

Throughout the paper, the data were presented as mean ± SEM (error bars). Statistical analyses were performed using R (v4.2.2) software. Data visualization was performed using Prism (v9.0) and R (v4.2.2) software. Unless otherwise stated, *p*-values for comparisons across two groups were performed using the Mann–Whitney test. False discovery rate (FDR) (Benjamini–Hochberg) was used to adjust the *p*-value for all multiple tests. Significance was defined as *p< 0.05, **p< 0.01, ***p < 0.001. Sample sizes and specific tests are denoted in the figure legends.

## Results

### 16S rRNA amplicon sequencing data summary

**Figure 1:**
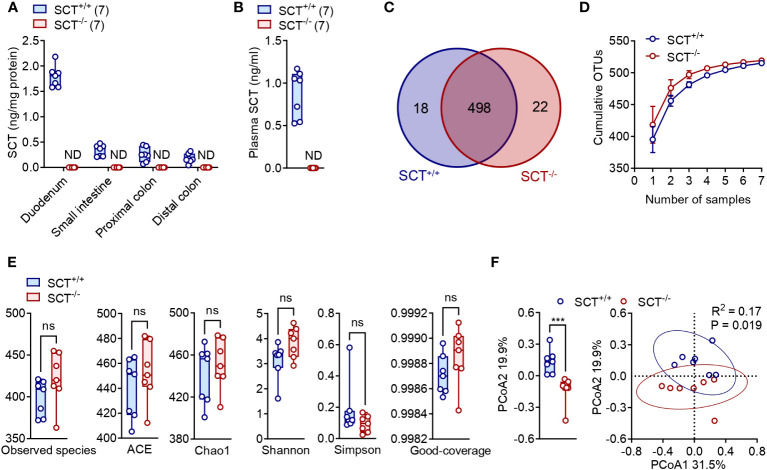
Alpha and Beta diversity of the gut microbiota in the SCT^+/+^ and SCT^-/-^ mice. **(A)** SCT concentrations in the duodenum, small intestine, proximal colon, and distal colon of SCT^+/+^ and SCT^-/-^ mice. **(B)** Plasma SCT levels of SCT^+/+^ and SCT^-/-^ mice fed *ad libitum*. **(C)** Venn diagram of OTUs between SCT^+/+^ and SCT^-/-^ groups. **(D)** OTUs accumulation curve plots of SCT^+/+^ and SCT^-/-^ groups. Error bars represent SD. **(E)** Comparison of 6 alpha diversity indexes. **(F)** PCoA plot of the gut microbiota using the Bray-Curtis distance metric (PERMANOVA). Each point represents the composition of the intestinal microbiota of one sample. The comparison at the PCoA2 level is given on the left. Data were presented as box plots with whiskers from minima to maxima, the central line at the 50th percentile, and the ends of the box at the 25th and 75th percentiles. Mann–Whitney test. ND, not detected. Ns, no significant; ***p < 0.001. Error bars represent SEM.

In this study, 16S rRNA amplicon sequencing was applied to reveal the differences in the gut microbiome between SCT^+/+^ (n = 7) and SCT^-/-^ (n = 7) mice. To show that SCT is not expressed in the SCT^-/-^ mice, RNA was extracted from the internal organs (brain, duodenum, heart, kidney, large intestine, liver, lung, small intestine, and stomach) of SCT^+/+^ and SCT^-/-^ mice and quantitative PCR was performed using specific SCT primers. We detected the expression of *Sct* mRNA in SCT^+/+^ mice, while *Sct* mRNA was not detected in all the tested tissues of SCT^-/-^ mice (**Supplementary Figure 1**). Consistently, hormone assay showed that SCT was detected in the duodenum, small intestine, colon, and plasma of SCT^+/+^, but not in SCT^-/-^ mice (**Figures 1A, B**). Together, these results indicate that SCT^+/+^ mice have normal SCT expression, while SCT^-/-^ mice are completely SCT deficient at both mRNA and protein levels.

A total of 952,511 clean paired reads were obtained from the raw data at a high data utilization rate (92.97 ± 0.32%), with an average of 67,574 ± 761 paired reads per sample for the SCT^+/+^ group and an average of 68,499 ± 119 paired reads per sample for the SCT^-/-^ group (**Supplementary Table 1**). High-quality reads were concatenated into consensus sequences (i.e., hypervariable region Tags), and then the spliced Tags were clustered into operational taxonomic units (OTUs) (**Supplementary Table 1**). The average OTU numbers obtained from SCT^+/+^ (398.9 ± 8.0) and SCT^-/-^ (421.6 ± 11.9) mice were not significantly different (**Supplementary Table 1**). SCT^+/+^ and SCT^-/-^ mice shared 498 common OTUs, with 18 and 22 unique OTUs, respectively (**Figure 1C**; **Supplementary Table 2**). To assess whether sequencing depth was sufficient to make stable estimates of species richness, we plotted OTUs rarefaction curves and showed that sequencing was saturated without increasing sample size. The sequencing depth covered all species in the sample, and more samples made little marginal contribution to new OTU discovery (**Figure 1D**).

### Gut microbial diversity between SCT^+/+^ and SCT^-/-^ mice

We then analyzed Observed species, ACE, Chao1, Shannon, Simpson, and Coverage as the six common alpha diversity indexes of the gut microbiota (**Supplementary Table 3**; **Figure 1E**). These alpha diversity indexes showed no significant difference in either the species richness or diversity of the gut microbiota between the SCT^+/+^ and SCT^-/-^ mice (**Figure 1E**). For beta diversity, principal coordinates analysis (PCoA) using Bray–Curtis distances generated from relative abundances of bacterial species was performed (PERMANOVA, R^2^ = 0.17, P = 0.019). The first principal axis (PCoA1) could explain 31.5% of the sample differences, and the second principal axis (PCoA2) could explain 19.9% of the sample differences (**Figure 1F**). Analysis revealed significant differences between SCT^+/+^ and SCT^-/-^ mice only at PCoA2 levels (**Figure 1F**). These findings indicate that lack of SCT barely alters gut microbiome diversity in adult male mice fed a standard chow diet.

### Taxonomic composition of the gut microbiota

**Figure 2:**
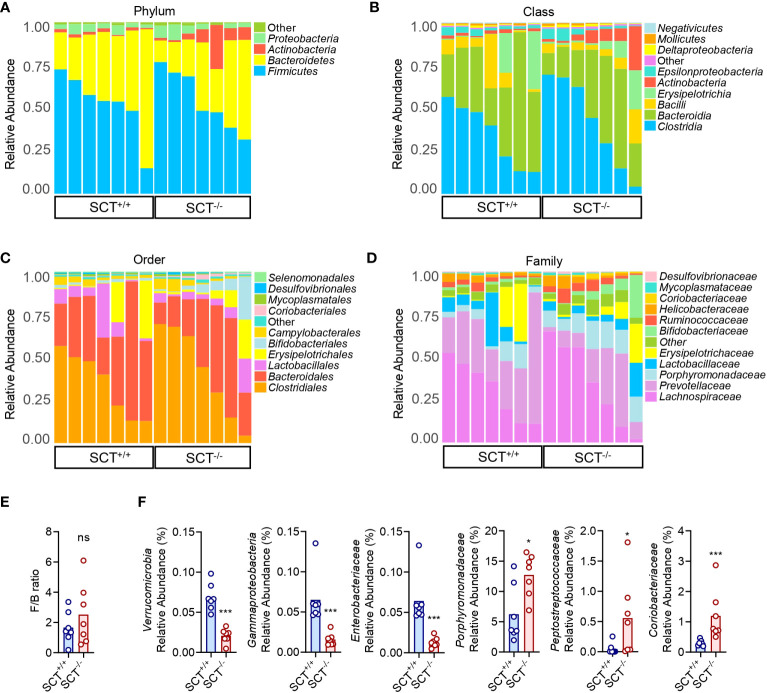
Taxonomic comparison of gut microbiota in SCT^+/+^ and SCT^-/-^ mice. Taxonomic composition of the gut microbiota of SCT^-/-^ mice (n = 7) and SCT^+/+^ controls (n = 7) at the phylum **(A)**, class **(B)**, order **(C)**, and family **(D)** levels. **(E)** The F/B ratio between the SCT^+/+^ and SCT^-/-^ groups. **(F)** Significantly changed bacterial taxa between the SCT^+/+^ and SCT^-/-^ groups. Components which an abundance below 0.5% in all samples were combined into “Other”. Mann–Whitney test. FDR was used to adjust the *p*-values in **(F)**. *p < 0.05; ***p < 0.001; ns, no significant. Error bars represent SEM.

The OTU representative sequences were taxonomically analyzed by the RDP classifier Bayesian algorithm, and the community composition of each sample at five (phylum, class, order, family, and genus) levels were calculated after annotation (**Supplementary Table 4**). At the phylum level, the relative abundance of the top four dominant phyla was not significantly different between SCT^+/+^ and SCT^-/-^ mice (**Figure 2A**; **Supplementary Figure 2A**). At the class level, *Clostridia*, *Bacteroidia*, *Bacilli*, *Erysipelotrichia*, and *Actinobacteria* dominated the gut microbiota in both SCT^+/+^ and SCT^-/-^ mice (**Figure 2B**; **Supplementary Figure 2B**). The major bacterial orders of the two groups were *Clostridiales*, *Bacteroidales*, *Lactobacillales*, *Erysipelotrichales*, and *Bifidobacteriales* (**Figure 2C**; **Supplementary Figure 2C**). At the family level, *Lachnospiraceae*, *Prevotellaceae*, *Porphyromonadaceae*, *Lactobacillaceae*, and *Erysipelotrichaceae* were the top 5 dominant families in the two groups (**Figure 2D**; **Supplementary Figure 2D**). There was no significant difference in Firmicutes/Bacteroidetes (F/B) ratios between the two groups (**Figure 2E**). Nevertheless, systemic knockout of SCT resulted in a decrease in the relative abundance of *Verrucomicrobia*, *Gammaproteobacteria*, and *Enterobacteriaceae* (**Figure 2F**). Conversely, *Porphyromonadaceae*, *Peptostreptococcaceae*, and *Coriobacteriaceae* were significantly enriched in SCT^-/-^ groups (**Figure 2F**). Overall, although the dominant microbiota was similar between the two groups, the absence of SCT altered the relative abundance of specific gut bacteria.

### The gut microbiota difference between SCT^+/+^ and SCT^-/-^ mice.

**Figure 3:**
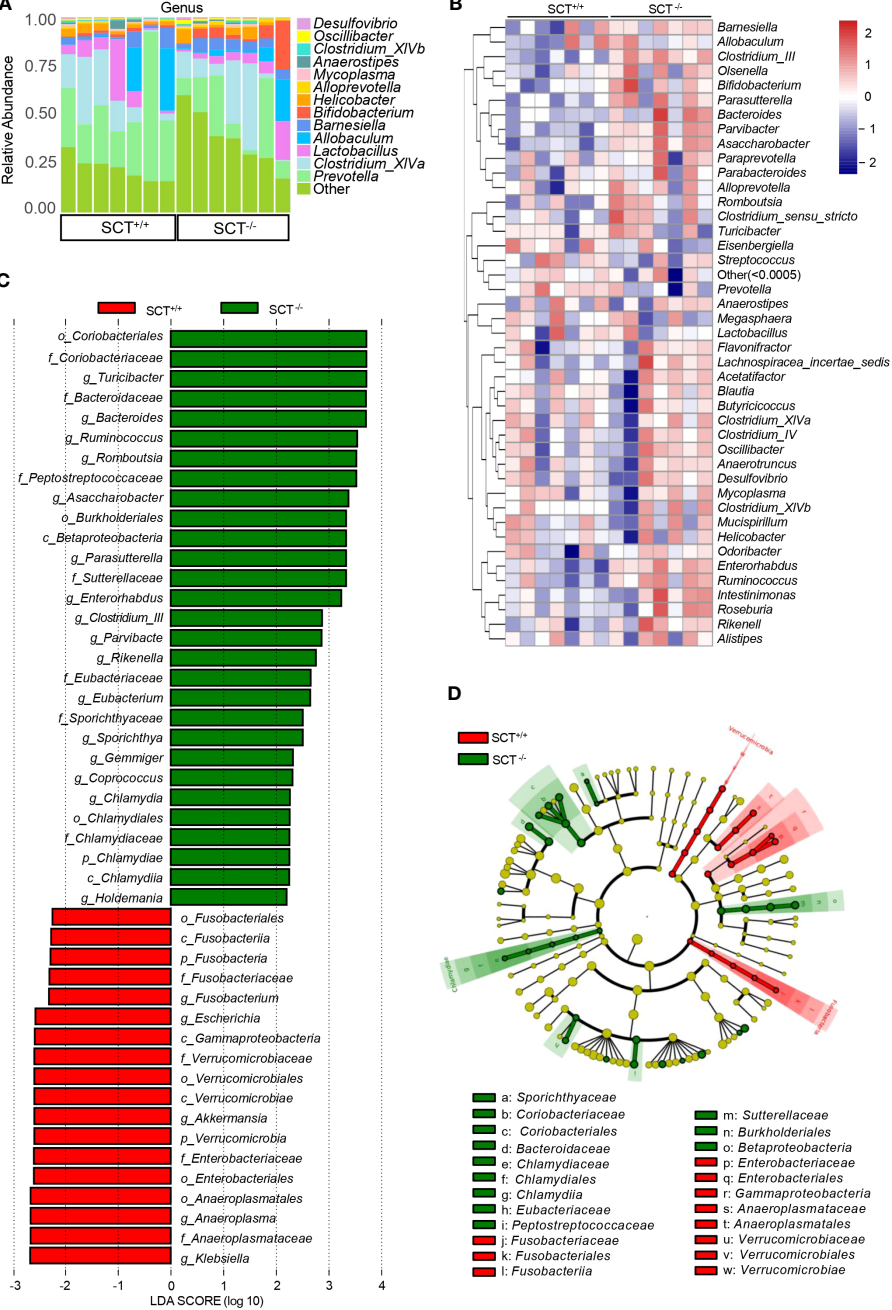
Analysis of the differences in gut microbiota between SCT^+/+^ and SCT^-/-^ mice. **(A)** The relative proportions of bacterial genera in SCT^-/-^ and SCT^+/+^ mice. Components which an abundance below 0.5% in all samples were combined into “Other”. **(B)** The genus-level abundance heat map. Relative abundance values are normalized through log_10_-transformation. **(C)** LEfSe bar plot represents the significantly differential taxa between SCT^+/+^ (red) and SCT^-/-^ (green) mice, based on effect size (LDAScore > 2). Enriched taxa in SCT^-/-^ mice (positive LDA score) and enriched taxa in SCT^++^ mice (negative LDA score). **(D)** LEfSe cladogram of the taxonomic differences in the gut microbiota between the two groups.

At the genus level, “Other” composed of low-abundance (<0.5%) bacteria has the highest proportion in the mouse gut microbiota, followed by *Prevotella*, *Clostridium_XlVa*, *Lactobacillus*, *Allobaculum*, and *Barnesiella* (**Figure 3A** and **Supplementary Figure 2E**). The relative abundance of *Asaccharobacter*, *Clostridium_III*, *Turicibacter*, *Enterorhabdus*, and *Parvibacter* was significantly higher in SCT^-/-^ mice than in SCT^+/+^ mice (**Figure 3B**; **Supplementary Table 5**). Conversely, the relative abundance of *Akkermansia* and *Escherichia* was significantly reduced in the SCT^-/-^ mice (**Figure 3B**; **Supplementary Table 5**). To further determine the effect of SCT deficiency on the specific gut microbial communities, a Linear discriminant (LDA) and effect size (LEfSe) analysis was performed to discover the different biomarkers in the microbiomes between groups (**Figures 3C, D**). A logarithmic LDA score cutoff of 2.0 was set to identify important taxonomic differences between the two groups. At the class level, *Fusobacteriia*, *Gammaproteobacteria*, and *Verrucomicrobiae* were enriched in the SCT^+/+^ mice, whereas *Betaproteobacteria* and *Chlamydiia* were enriched in the SCT^-/-^ mice (**Figures 3C, D**). At the order level, *Fusobacteriales*, *Verrucomicrobiales*, *Enterobacteriales*, and *Anaeroplasmatales* were enriched in the SCT^+/+^ mice, and *Coriobacteriales*, *Burkholderiales*, and *Chlamydiales* were enriched in the SCT^-/-^ mice (**Figures 3C, D**). At the genus level, *Fusobacterium*, *Escherichia*, *Akkermansia*, *Anaeroplasma*, and *Klebsiella* were enriched in the SCT^+/+^ mice, while *Turicibacter*, *Bacteroides*, *Ruminococcus*, *Romboutsia*, *Asaccharobacter*, *Parasutterella*, and *Enterorhabdus*, *etc.* were enriched in the SCT^-/-^ mice (**Figures 3C, D**). Taken together, these results suggest that systemic SCT deletion alters the enrichment of the specific gut bacteria in mice.

### Functional differences in the gut microbiota between SCT^+/+^ and SCT^-/-^ mice

**Figure 4:**
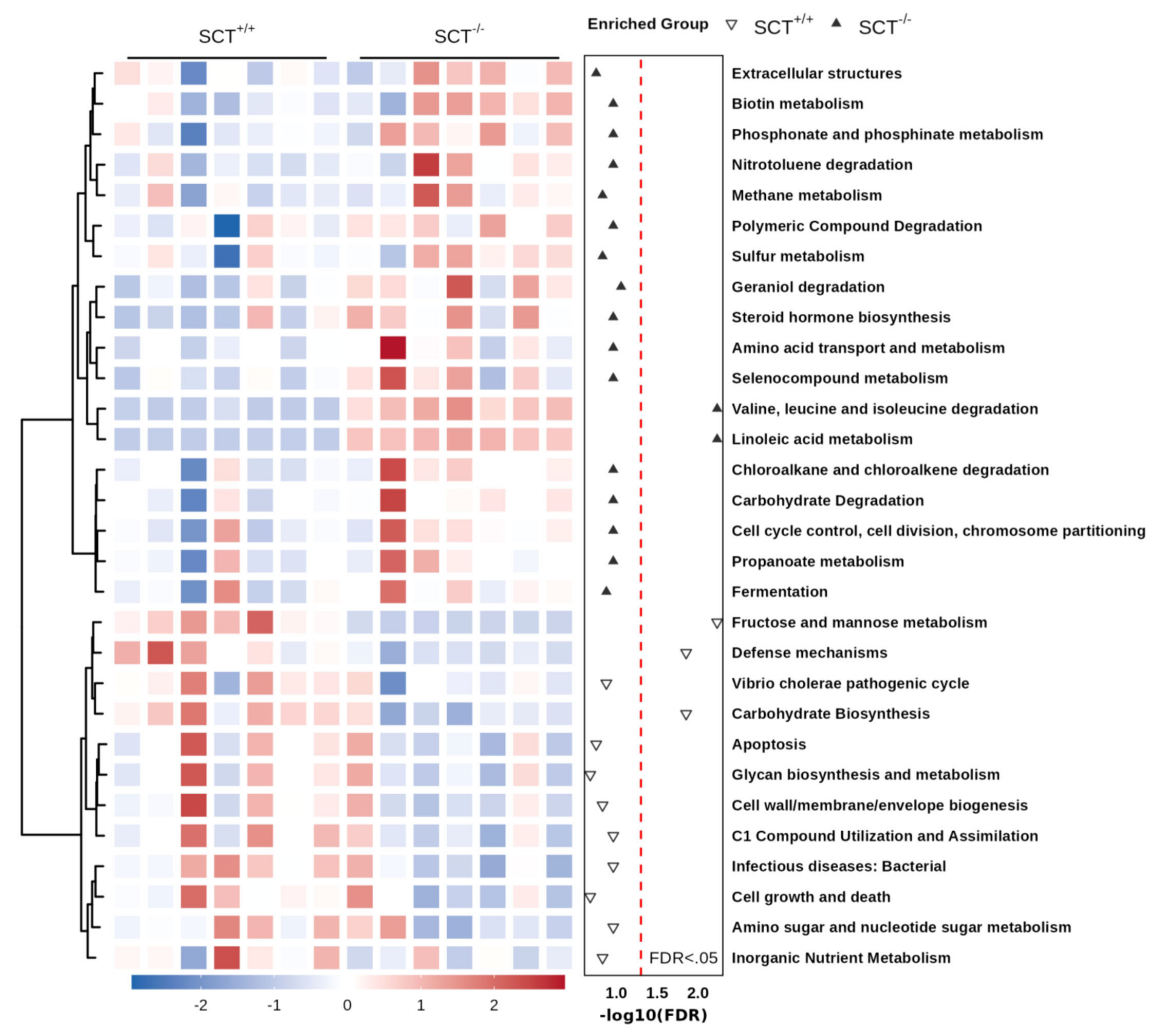
Functional prediction analysis of the mouse gut microbiota based on the KEGG database. The heat map of the top 30 KEGG pathways. Two-group comparison was performed using Mann–Whitney test. FDR (Benjamini–Hochberg) was used to adjust the *p*-values.

The concentration of fecal lipids and SCFAs of SCT^+/+^ and SCT^-/-^ mice was measured in this study. Fecal lipid profile results showed that fecal FFA, triglyceride, and total cholesterol levels were similar between genotypes (**Supplementary Figure 3**). In addition, GC-MS analysis did not reveal any changes in the SCFAs levels in feces, including acetate, propionate, butyrate, valerate, and isobutyrate (**Supplementary Figure 3**). To further investigate the changes in metabolic functions of the gut microbiota associated with SCT deficiency, we performed functional prediction analysis based on the KEGG database using PICRUSt2 (Langille et al., 2013). Difference analysis of the KEGG pathways showed that the branched-chain amino acid (valine, leucine, and isoleucine) degradation and linoleic acid metabolism are enriched in the SCT^-/-^ mice (**Figure 4**; **Supplementary Table 6**). On the other hand, compared with SCT^+/+^ mice, SCT deficiency resulted in decreased abundance of the following 3 pathways, including fructose and mannose metabolism, defense mechanisms, and carbohydrate biosynthesis (**Figure 4**; **Supplementary Table 6**). These results suggest that systemic SCT deletion induces potential changes in the specific metabolic pathways in the mouse gut microbiota.

## Discussion

Despite recent studies suggesting that GI hormones can be involved in regulating gut microbiota homeostasis (Sun et al., 2020), information about how SCT affects the composition and function of gut microbiota is scarce. In this study, we characterized the gut microbiome in fecal samples from homozygous SCT gene-null mice using 16S rRNA amplicon sequencing. We found that whole-body SCT deficiency did not significantly alter the quantity and species of OTUs of the gut microbiota in mice. However, based on the Bray-Curtis distance matrices, the PCoA showed that the community structure of the gut microbiota of SCT- deficient and wild-type control mice clustered separately, suggesting that systemic deletion of the SCT gene altered the structure of the gut microbiota.

*Firmicutes*, *Bacteroidetes*, *Actinobacteria*, *Proteobacteria*, and *Verrucobacteria* are the five major bacterial phyla that inhabit the gut of healthy humans and mice (Qin et al., 2010; Li et al., 2019; Magne et al., 2020; Ghosh and Pramanik, 2021). Among them, *Firmicutes* and *Bacteroidetes* accounted for more than 90% of the entire community (Rinninella et al., 2019; Magne et al., 2020). *Firmicutes* degrade polysaccharides through multienzyme complexes and protect colon health by producing butyrate from carbohydrates (Wexler and Goodman, 2017; Parada Venegas et al., 2019). *Bacteroides* assist the host in absorbing nutrients from the diet by fermenting dietary fiber (Zafar and Saier, 2021). The relative abundances of *Firmicutes* and *Bacteroidetes* did not change between SCT^+/+^ and SCT^-/-^ mice. Notably, the F/B ratio was considered to be positively correlated with body weight. Existing evidence suggest that a high F/B ratio increases energy absorption efficiency, leading to obesity and related dysfunction (Ley et al., 2006; Murphy et al., 2010). Conversely, a low F/B ratio was associated with a lean phenotype, cardiovascular health, and a balanced immune system, generally considered to be beneficial for health (Nicholson et al., 2012; Tang et al., 2017). In this study, the F/B ratio of SCT^-/-^ mice was not significantly different from that of SCT^+/+^ mice. This echo previous studies in which either SCT or SCT receptor knockout mice fed a standard rodent diet exhibited normal body weight (Yamagata et al., 2008; Sekar and Chow, 2014).

At the genus level, the enrichment of *Akkermansia* and *Escherichia*, which normally colonize the healthy gut microbiota (Tap et al., 2009; Turnbaugh et al., 2009; Wang et al., 2019), was downregulated in SCT^-/-^ mice. These bacteria are generally harmless to the host and may exert beneficial effects. Among them, *Akkermansia*, as a recently discovered promising probiotic (Zhai et al., 2019; Zhang et al., 2019b), is known to be associated with metabolic regulation and immune responses (Plovier et al., 2017; Routy et al., 2018). Metagenomic studies have shown that humans and mice with chronic intestinal inflammation, such as inflammatory bowel disease (IBD), have a lower abundance of *Akkermansia* in the gut (Rodrigues et al., 2022). There is evidence that the transgenic ablation of SCT-expressing cells leads to the sudden onset of colitis (Rindi et al., 2004). On the other hand, colonic endogenous SCT may interact with oxytocin to prevent IBD (Welch et al., 2010). Moreover, SCT may be involved in the immune response of colonic microbes to prevent tissue damage caused by excessive inflammation (Welch et al., 2010). Taken together, the reduced abundance of *Akkermansia* may be a sign of abnormalities in the intestinal immune system of SCT^-/-^ mice, which warrants further investigation.

The main producers of SCFAs in the healthy gut, such as *Lachnospiraceae*, *Ruminococcaceae*, and *Lactobacillaceae* (Herrmann et al., 2018) were not altered by SCT deficiency. It is therefore not surprising that fecal SCFAs levels were unchanged in SCT^-/-^ mice. Notably, the latest study showed that ablation of enteroendocrine cells, including S cells, also did not change fecal SCFAs levels (Blot et al., 2023). Bolt et al. found that loss of enteroendocrine cells triggers rapid remodeling of the gut microbiota and adaptive metabolic responses, thereby activating alternative metabolic pathways to adjust energy utilization (Blot et al., 2023). However, unlike ablation of enteroendocrine cells, which resulted in drastic changes in metabolic pathways predicted by PICRUSt2 (Blot et al., 2023), lack of SCT resulted in only mild changes in predicted metabolic pathways. Thus, the impact of SCT deficiency on metabolites of mouse gut microbiota may be limited. In fact, our previous study showed that plasma lipid and glucose levels were not altered in standard chow diet-fed SCT receptor knockout mice (Sekar and Chow, 2014), and the present data also showed that fecal lipid levels did not change in SCT-deficient mice. Overall, 16S rRNA amplicon sequencing demonstrates that systemic SCT knockout has minor effects on the composition and function of the gut microbiota in adult male mice fed a standard chow diet. Nonetheless, changes in specific microbes suggest a potential link between SCT and the gut microbiota, and this information may help to understand the role of SCT in GI homeostasis from a microbial perspective.

## Data availability statement

The original contributions presented in the study are publicly available. This data can be found here: NCBI PRJNA993811.

## Ethics statement

The animal study was approved by the Committee on the Use of Live Animals in Teaching and Research (CULATR) of the University of Hong Kong. The study was conducted in accordance with the local legislation and institutional requirements.

## Author contributions

FZ: Conceptualization, Data curation, Formal analysis, Investigation, Methodology, Project administration, Resources, Software, Supervision, Validation, Visualization, Writing – original draft, Writing – review & editing. ZT: Data curation, Methodology, Writing – review & editing. CC: Data curation, Formal analysis, Methodology, Visualization, Writing – review & editing. BC: Conceptualization, Funding acquisition, Project administration, Supervision, Writing – review & editing.

## Supplementary material

Supplementary Figure 1 The relative expression levels of *Sct* mRNA in SCT^+/+^ and SCT^-/-^ mice. *Sct* mRNA expression in the brain, duodenum, heart, kidney, colon, liver, lung, small intestine, and stomach of SCT^+/+^ and SCT^-/-^ mice. ND, not detected. Error bars represent SEM.

Supplementary Figure 2 Taxonomic analysis of the gut microbial community composition of SCT^+/+^ and SCT^-/-^ mice. Relative abundance of the gut microbiota components of each group at the level of **(A)** phylum, **(B)** class, **(C)** order, **(D)** family, and **(E)** genus. Components which an abundance below 0.5% in all samples were combined into Other. Error bars represent SEM.

Supplementary Figure 3 Analysis of fecal lipids and SCFAs. **(A–C)** Fecal lipids: **(A)** FFA, **(B)** triglyceride, and **(C)** total cholesterol of SCT^+/+^ (n = 10) and SCT^-/-^ (n = 10) mice. **(D–H)** Fecal SCFAs: **(D)** acetate, **(E)** propionate, **(F)** butyrate, **(G)** valerate, and **(H)** isobutyrate of SCT^+/+^ (n = 10) and SCT^-/-^ (n = 10) mice. Data were presented as box plots with whiskers from minima to maxima, the central line at the 50th percentile, and the ends of the box at the 25th and 75th percentiles. Mann–Whitney test. Ns, no significant. Error bars represent SEM.
