# Supplementary figures and images for: Effects of secretin gene knockout on the diversity, composition, and function of gut microbiota in adult male mice
Source: Front Cell Infect Microbiol. 2023 Dec 13;13:1257857. doi: 10.3389/fcimb.2023.1257857 (PMC10753818; doi:10.3389/fcimb.2023.1257857)

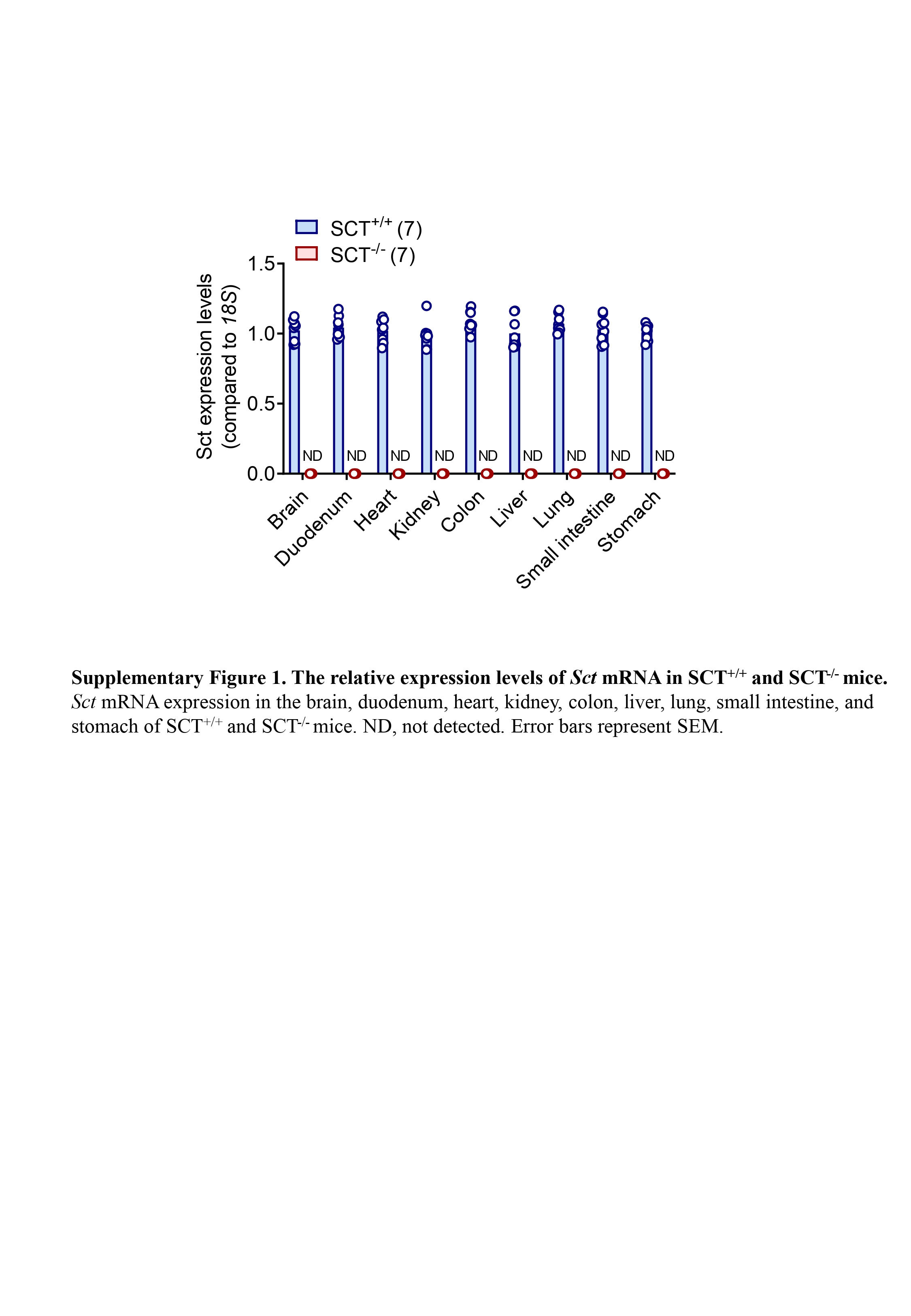

Supplement: Supplementary Figure 1 — The relative expression levels of Sct mRNA in SCT+/+ and SCT-/- mice. Sct mRNA expression in the brain, duodenum, heart, kidney, colon, liver, lung, small intestine, and stomach of SCT+/+ and SCT-/- mice. ND, not detected. Error bars represent SEM. [file Image_1.jpeg]

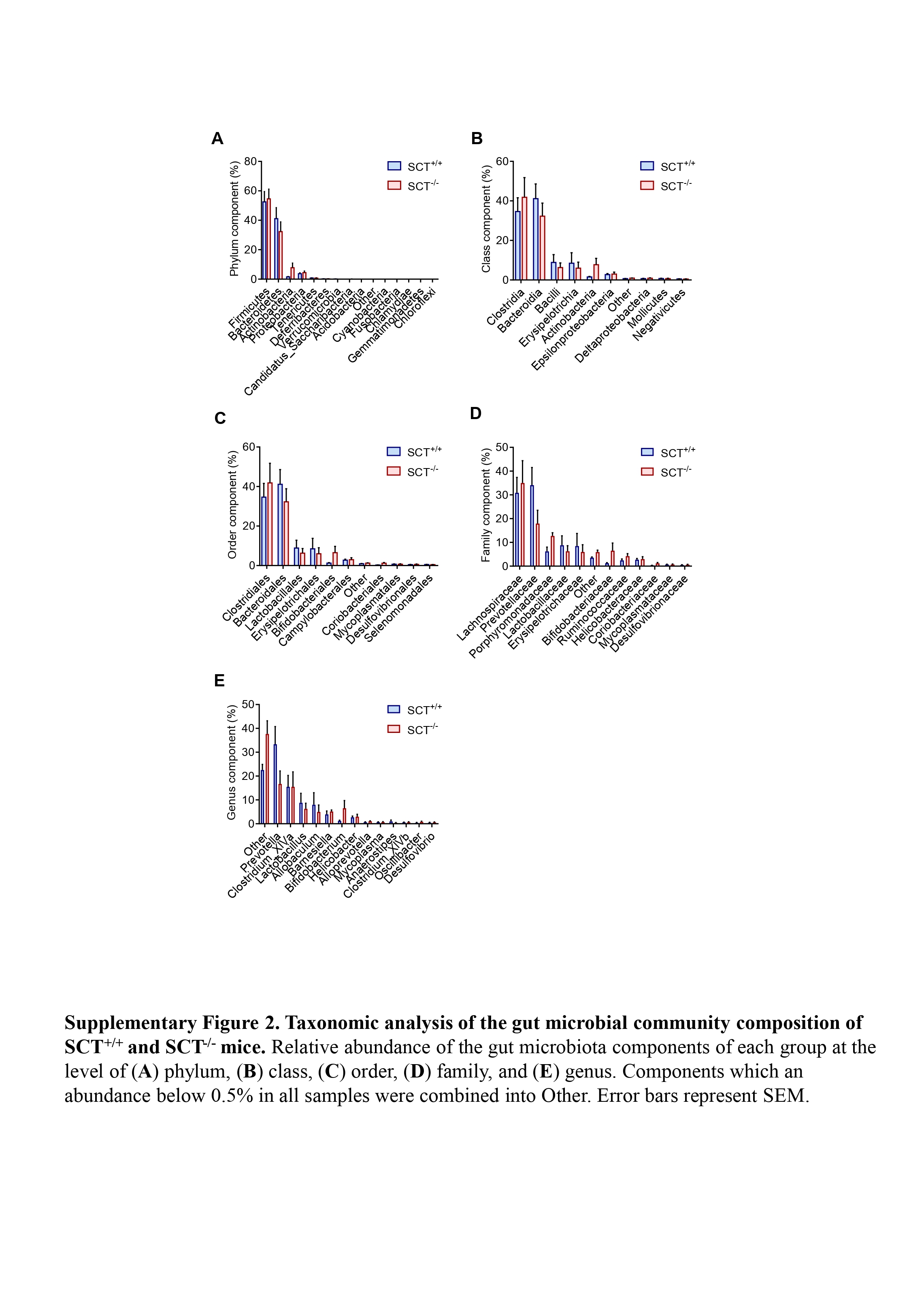

Supplement: Supplementary Figure 2 — Taxonomic analysis of the gut microbial community composition of SCT+/+ and SCT-/- mice. Relative abundance of the gut microbiota components of each group at the level of (A) phylum, (B) class, (C) order, (D) family, and (E) genus. Components which an abundance below 0.5% in all samples were combined into Other. Error bars represent SEM. [file Image_2.jpeg]

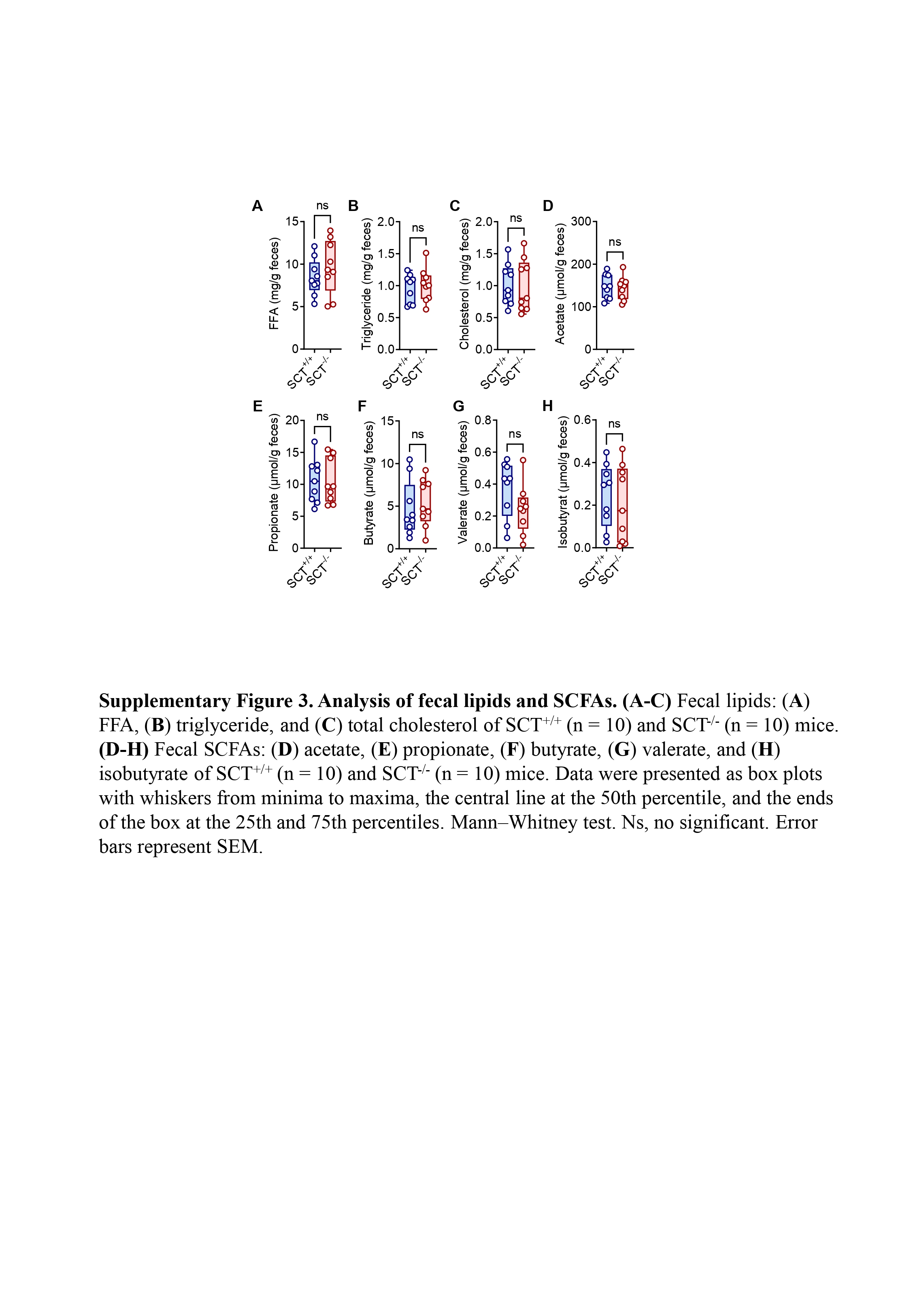

Supplement: Supplementary Figure 3 — Analysis of fecal lipids and SCFAs. (A–C) Fecal lipids: (A) FFA, (B) triglyceride, and (C) total cholesterol of SCT+/+ (n = 10) and SCT-/- (n = 10) mice. (D–H) Fecal SCFAs: (D) acetate, (E) propionate, (F) butyrate, (G) valerate, and (H) isobutyrate of SCT+/+ (n = 10) and SCT-/- (n = 10) mice. Data were presented as box plots with whiskers from minima to maxima, the central line at the 50th percentile, and the ends of the box at the 25th and 75th percentiles. Mann–Whitney test. Ns, no significant. Error bars represent SEM. [file Image_3.jpeg]
